# An Assessment of the Chondroprotective Effects of Intra-Articular Application of Statin and Tetracycline on Early-Stage Experimental Osteoarthritis

**DOI:** 10.5402/2012/182097

**Published:** 2012-05-29

**Authors:** Mustafa Dinc, Muhammed Sadik Bilgen, Abdullah Kucukalp, Omer Faruk Bilgen

**Affiliations:** ^1^Department of Orthopaedics and Traumatology, Nizip State Hospital, Gaziantep, Turkey; ^2^Ortopedi ve Travmatoloji Bölümü, Tıp Fakültesi, Uludağ Üniversitesi, 16059, Görükle Kampüsü, Bursa, Turkey

## Abstract

*Objectives*. To compare the effects of intra-articular application of statin and tetracyclines on cartilage and synovial tissue on experimental osteoarthritis. *Methods*. Osteoarthritis was created in 30 rabbits of 3 groups. The control group received saline intra-articularly, statin group, atorvastatin and the tetracycline group, doxycycline once a week for 3 weeks. Chondral and synovial tissues were evaluated macroscopically and histopathologically. *Results*. Macroscopic evaluation determined mean values of control group 3.0, statin group 0.56, and tetracycline group 2.5. Histopathological evaluations determined mean values; femoral medial condyle cartilage tissue, control group, 14.60 ± 1.00, statin group 2.20 ± 1.30, tetracycline group 12.7 ± 5.39: tibia medial plateau, control group, 14.33 ± 8.68, statin group 2.89 ± 1.96, tetracycline group, 15.90 ± 7.03: synovial tissue, control group 12.22 ± 3.63, statin group 4.33 ± 2.69, tetracycline group 10.70 ± 2.62. Average values of synovial tissue cell layer thickness were control group 14.46 ± 2.35 *μ*m, statin group 10.56 ± 1.01 *μ*m, tetracycline group 12.80 ± 0.79 *μ*m. All measurements showed statistically significant differences between statin and control groups (*P* < 0.05) but not between tetracycline and control groups (*P* > 0.05). *Conclusions*. Tetracycline has little effect due to chemical modification requirement, and the effect is dose dependent. Statins have chondroprotective effects, so may become a novel therapeutic agent in osteoarthritis management after chemical processing.

## 1. Introduction

Osteoarthritis (OA) is the most frequently encountered form of arthritis in which a slow and progressive deterioration of the cartilage tissue particularly in weight-bearing joints is seen. This disease is characterised pathologically by the destruction of the cartilage in the joint, the formation of new bone around the joint (osteophytes), changes in the subchondral bone, and synovitis and fibrosis in the joint capsule [1, 2]. Several different factors have been described which influence the impairment of the homeostasis (anabolic and catabolic balance) between the cells and the extracellular matrix in the pathogenesis of OA. [2–4]. Produced from chondrocytes in the cartilage tissue and synovial cells in the synovial tissue, the stimulating signal formed on the catabolic effect of cytokines such as tumour necrosis factor alpha (TNF-*α*), interleukine-1 (IL-1), interleukine-6 (IL-6), and nitric oxide (NO) has been shown to activate matrix metalloproteinases (MMP) such as collagenase, gelatinase, aggrecanase, elastase, and fibronectin destructive stromelysin-1 [3–6].

 In recent years, with the understanding of OA pathophysiology, there has been work on the development of pharmacological agents to halt the cartilage destruction and slow its progression. Many studies have shown the anti-inflammatory and immunomodulator pleiotropic effects on connective tissue of the antibiotic tetracycline and statins as a lipid-reducing agent [7–14]. It has been thought that these agents may have protective effects on cartilage and there are a limited number of studies specifically on cartilage connective tissue [10, 15–20].

When the pathophysiology of OA is considered, the hypothesis was proposed that the pleiotropic effects of statins and tetracyclines may have an effect on the stimulating cytokines which play a role in the development of OA, and on the release of proteolytic enzymes responsible for degeneration. Thinking that these two medications may have chondroprotective effects on cartilage as a specific connective tissue, there have recently been several experimental animal model studies researching the effects of these medications on early-stage OA [5, 10, 15, 17–20].

 This experimental study aimed to research the chondroprotective effects of intraarticular administration of statin and tetracycline on the cartilage and synovial tissue of rabbits in which early-stage OA had been developed.

## 2. Materials and Methods

Approval for the study was obtained from Uludag University Medical Faculty Experimental Animal Research Ethics Committee. Thirty adult white New Zealand rabbits weighing 2500–4000 gr, obtained from the Experimental Animal Breeding and Research Centre, were used in the study. The experimental model of knee OA, was created by cutting the anterior cruciate ligament (ACL) of the rabbits as described by Yoshioka et al. [21]. 

For the administration of the intra-articular injection, the rabbits were randomly allocated into 3 groups. The control group received 0.5 mL physiological saline, the statin group received 0.4 mg/mL/kg of the mixture obtained from adding 40 mg atorvastatin to 100 mL of physiological saline, and the tetracycline group received 1 mg/mL/kg of the mixture obtained from adding 100 mg doxycycline to 100 mL of physiological saline.

### 2.1. Surgical Technique

As infection prophylaxis, 50 mg/kg cefazolin sodium was administered intramuscularly 30 minutes prior to the surgical intervention. Anaesthesia was administered intramuscularly at dosages of 2% xylazine hydrochloride 8 mg/kg and ketamine hydrochloride 100 mg/kg. The surgical site of the shaved right back leg of the rabbits was cleaned with 10% povidone-iodine solution then sterile draped. After a longitudinal anterior midline skin incision, an adequate opening was obtained by medial parapatellar arthrotomy with the patella dislocated laterally. The ACL was cut with the knee in full flexion. The anterior drawer test confirmed that the ACL had been completely cut. The joint cavity was washed with saline and the joint capsule sutured with 3/0 absorbable sutures and the skin was closed with 3/0 nonabsorbable sutures. Postoperative analgesia was administered as 1-2 mg/kg 100 mL paracetamol in the drinking water then the rabbits were left active in cages with standard feed.

While sedated with 30 mg/kg ketamine hydrochloride, the rabbits were randomly allocated into 3 groups of 10 for the intraarticular injections. From the first week postoperatively, once a week for 3 weeks, the control group received 0.5 mL saline solution, the statin group received 0.4 mg/kg of 40 mg atorvastatin in 100 mL saline and the tetracycline group received 1 mg/mL/kg of 100 mg doxycycline in 100 mL saline. Twelve weeks after the final injection the experimental animals were sacrificed by decapitation. To develop an experimental OA model, the knee joints were removed including the synovial, femoral, and tibial joint surfaces. The medial compartment of the knee joints (medial femoral condyle and tibial medial plateau) were evaluated using the morphological stage system described by Pelletier et al. [22], to determine the depth of cartilage tissue erosion and degenerative changes in respect of the presence of surface and mid layer erosion, deep layer erosion, and subchondral bone erosion and minimal fibrillation.

### 2.2. Tissue Preparation

The tissue samples taken from the experimental animals, after being separated from surrounding soft tissue, were kept in 10% formaldehyde solution for 5 days for histopathological examination. Bone tissues were kept in 10% formic acid solution until decalcification. The samples were then dehydrated and fixed in paraffin blocks. After routine monitoring, 5 micron thickness sagittal sections were stained with haematoxylin-eosin and safranin-O for histopathological evaluation.

### 2.3. Histopathological Evaluation

Four layers of cartilage tissue were examined to evaluate histological and histochemical changes. Using the joint cartilage lesion histological and histochemical stages system defined by Mankin et al. [23] and modified by Yoshimi et al. [24], the joint cartilage tissue was evaluated for cartilage tissue layer histological structure changes, cellular changes, safranin-O involvement, tidemark structure entirety, and pannus formation. The points obtained were statistically compared between the groups.

In the histological evaluation of synovial tissue changes, the classification system used was that defined by Yoshimi et al. [24] formed of two main parts. In this classification system, the first part examines cell hypertrophy, hyperplasia, and inflammatory cell infiltration of the cellular layer covering the synovia and the second part evaluates granulation tissue proliferation formed in the subsynovial area, vascularisation, and inflammatory cell infiltration. The points obtained from both parts were totaled for each of the three groups and statistically compared. The results of the evaluation of the synovial tissue with the measurements of the synovial cell layer thickness in microns for the three groups were statistically compared.

### 2.4. Statistical Evaluation

The data obtained were recorded on computer with SPSS for Windows 13.0. The variables related to macroscopic and microscopic evaluations were given as mean and standard deviations. The result values were compared using Mann-Whitney *U* test. A *P* value of <0.05 was accepted as statistically significant.

## 3. Results

During the study one rabbit in the control group died from respiratory problems and one in the statin group died from septic arthritis leaving a total of 28 rabbits for the experiment.

**Figure 1:**
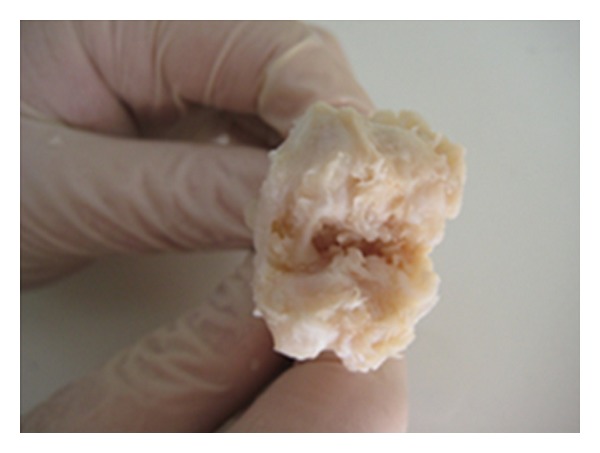
Stage 4 cartilage damage in the macroscopic evaluation in the control group.

**Figure 2:**
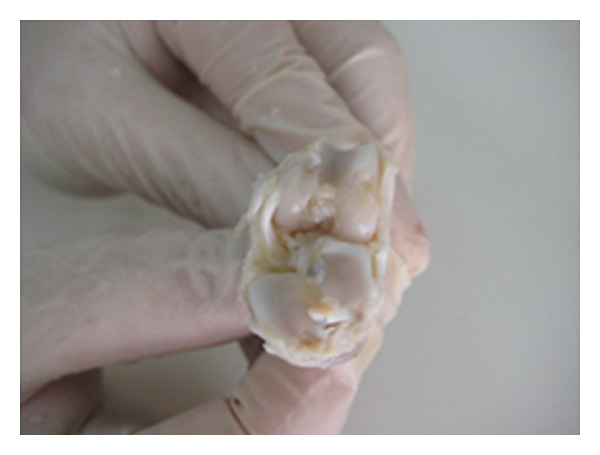
Stage 0 cartilage damage in the macroscopic evaluation of the statin group.

 When the lesions formed in the cartilage tissue of the medial compartment of the knee were macroscopically evaluated, the degenerative changes of the rabbits in the control group were determined as 2 (22%) at stage 2, 5 (55%) at stage 3, and 2 (22%) at stage 4. In the statin group, degenerative changes were determined as 5 (55%) at stage 0, 3(33%) at stage 1, and 1 (11%) at stage 2. In the tetracycline group degenerative changes were determined as 5 (50%) at stage 2 and 5 (50%) at stage 3. Advanced stage cartilage damage, deep layer, and subchondral bone erosion were observed in 7(77%) rabbits in the control group and 5 (50%) in the tetracycline group and no advanced stage degenerative changes were determined in the statin group (0%). Statistical evaluation of these results showed a statistically significant difference between the control group and the statin group (*P* < 0.05) (Figures 1 and 2) and no statistically significant difference between the control group and the tetracycline group (*P* > 0.05). 

**Figure 3:**
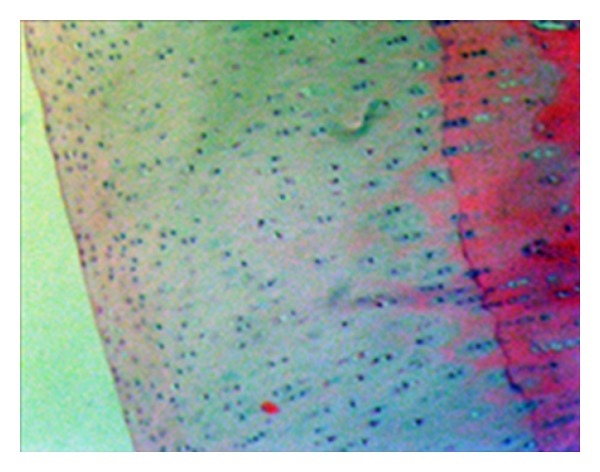
Normal joint cartilage histological view (statin group—stage 0 damage) (HE, ×200).

**Figure 4:**
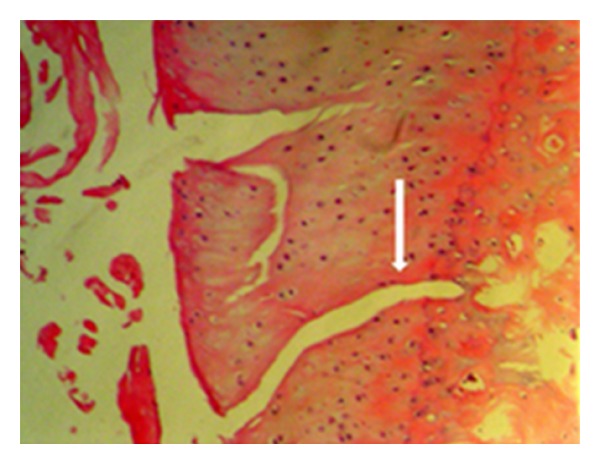
Cleavage extending to the radial area shown by the white arrow (control group—stage 4 damage) (HE, ×200).

**Figure 5:**
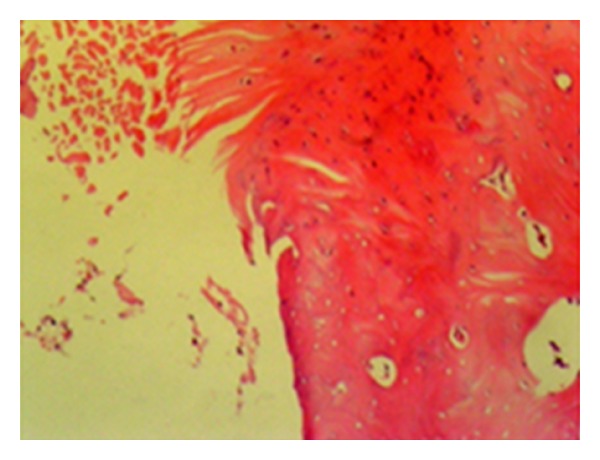
Total disorganisation of the joint cartilage (control group—stage 3 damage) (HE, ×200).

**Figure 6:**
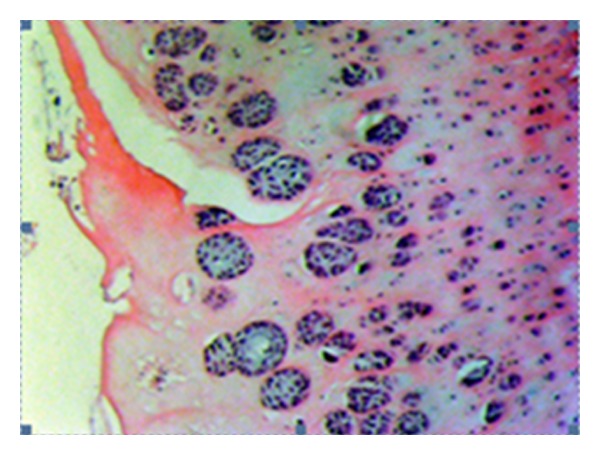
Serious cloning of the chondrocytes in the transitional and radial layers (tetracycline group—3 damage) (HE, ×200).

**Figure 7:**
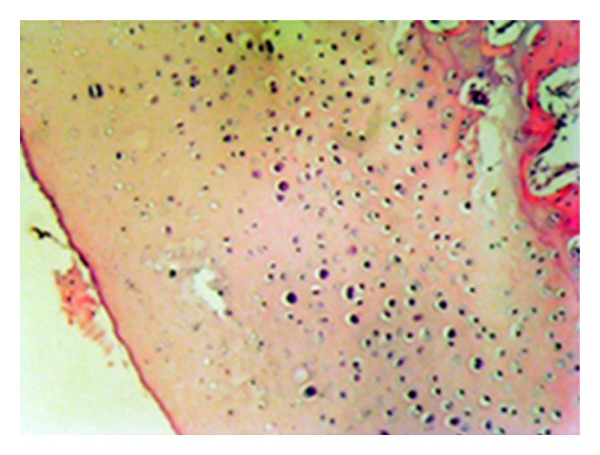
Mid level hypocellularity in the chondrocytes of the transitional and radial layers (tetracycline group—stage 3 damage) (HE, ×200).

**Figure 8:**
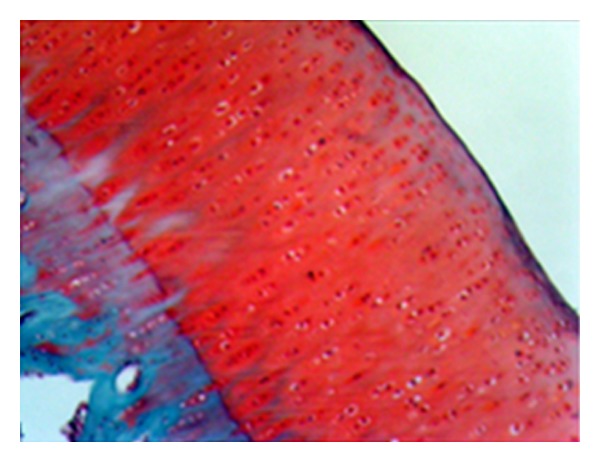
Safranin-O involvement in normal joint cartilage (statin group—stage 0 damage) (Safranin—O, ×200).

**Figure 9:**
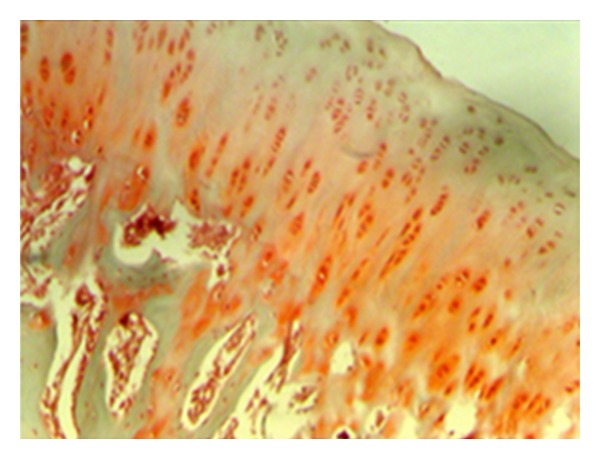
Serious loss of safranin-O involvement (control group—stage 4 damage) (Safranin-O, ×200).

**Figure 10:**
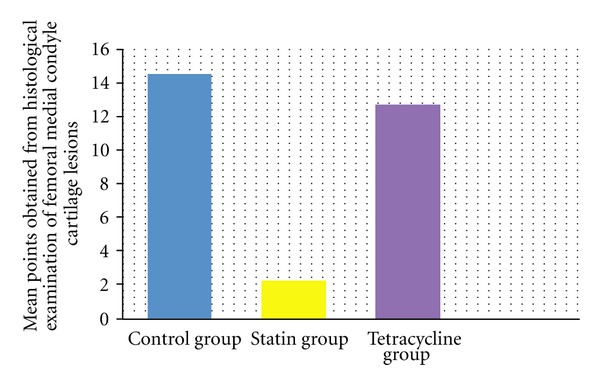
Comparison of mean points obtained from histological examination of the femoral medial condyle cartilage tissue lesions. In the statin group, compared to the control group and tetracycline group, a statistically significant difference was determined (*P* < 0.05) in normal and mild surface disorganisation of medial compartment cartilage tissue, cell structure near to normal in the cartilage layers, normal safranin-O involvement, intact tidemark structure, and pannus formation were determined. The difference between the tetracycline group and the control group was not statistically significant (*P* > 0.05). The results are given as mean and standard deviation.

**Figure 11:**
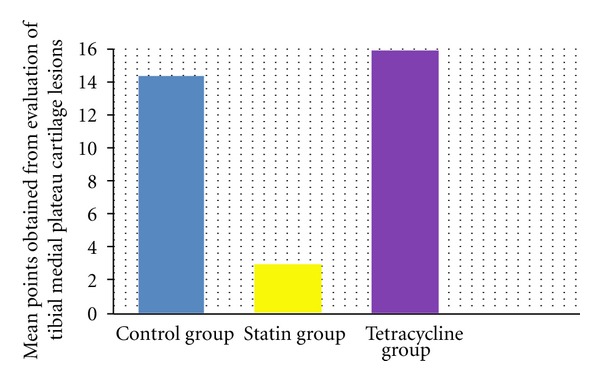
Comparison of mean points obtained from histological examination of the medial plateau cartilage tissue lesions. In the statin group, compared to the control group and tetracycline group, a statictially significant difference was determined (*P* < 0.05) in normal and mild surface disorganisation of medial compartment cartilage tissue, cell structure near to normal in the cartilage layers, normal safranin-O involvement, intact tidemark structure and pannus formation were determined. The difference between the tetracycline group and the control group was not statistically significant (*P* > 0.05). The results are given as mean and standard deviation.

According to the modified Mankin classification system used in the histological and histochemical evaluation of the cartilage tissue lesions in the medial compartment of the knee, the results of the examination of cartilage structure, cellular changes in the tangential, transitional and radial layers, safranin-O involvement, impairment of the tidemark structure, and pannus formation were determined as total points obtained from the evaluation of lesions developed in the femoral medial condyle, control group mean 14.56 ± 1.00, statin group mean 2.2 ± 1.30, and tetracycline group mean 12.70 ± 5.39 and total points of the tibia medial plateau cartilage tissue for the three groups were mean 14.33 ± 8.68, 2.89 ± 1.96, and 15.90 ± 7.03, respectively (Figures 3, 4, 5, 6, 7, 8, and 9). Statistical comparison of the points obtained for femoral medial condyle and tibia medial plateau cartilage tissue determined a statistically significant difference between the control group and the statin group (*P* < 0.05) and no difference was observed between the control group and the tetracycline group (*P* > 0.05). A comparison between the statin group and the tetracycline determined a statistically significant difference in favour of the statin group (*P* < 0.05) (Figures 10 and 11).

**Figure 12:**
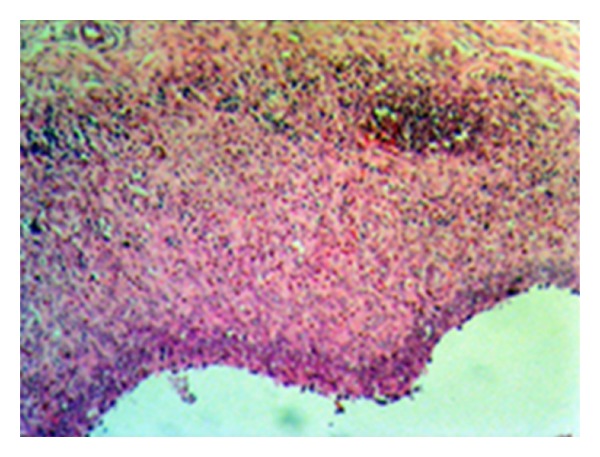
Mid-level hyperplasia and hypertrophy in the cell layer covering the synovia and serious inflammatory cell infiltration in the subsynovial area (control group—stage 4 damage) (HE, ×100).

**Figure 13:**
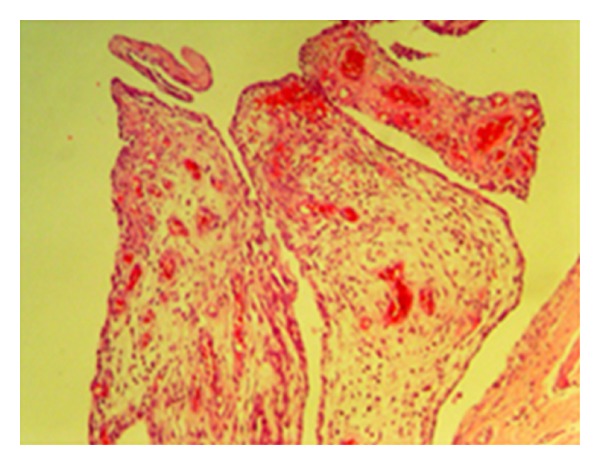
Serious vascularisation in the subsynovial area (control group—stage 4) (HE, ×100).

**Figure 14:**
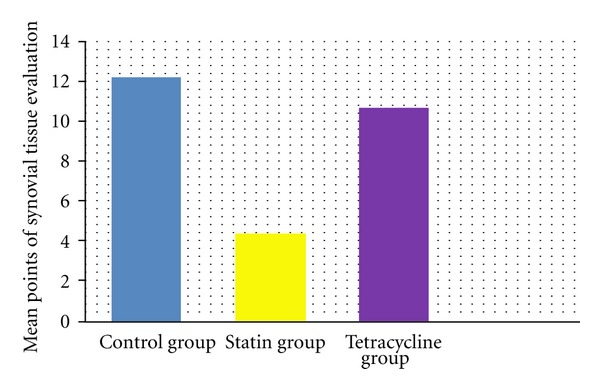
Comparison of mean points obtained from the histological evaluation of synovial tissue. Compared to the control group and tetracycline group, in the statine group a statistically significant difference was determined in reduced cell hyperplasia, hypertroph, and inflammatory cell infiltration, in the cell layer covering the synovia and proliferation of granulation tissue, vascularisation, and reduced inflammatory cell infiltration in the subsynovial area (*P* < 0.05). No difference was determined between the tetracycline group and the control group (*P* > 0.05). The results are given as mean points with standard deviation.

 The total points obtained from histological evaluation of synovial tissue changes of cell hypertrophy, hyperplasia of the cellular layer covering the synovia, inflammatory cell infiltration and subsynovial area granulation tissue proliferation, vascularisation, and inflammatory cell infiltration, were determined as 12.22 ± 3.63 in the control group, 4.33 ± 2.69 in the statin group and 10.70 ± 2.62 in the tetracycline group (Figures 12 and 13). Statistical comparison of the points obtained determined a statistically significant difference between the control group and the statin group (*P* < 0.05) and no difference was observed between the control group and the tetracycline group (*P* > 0.05). A comparison between the statin group and the tetracycline determined a statistically significant difference in favour of the statin group (*P* < 0.05) (Figure 14).

 The synovial cell layer thickness was measured as mean 14.56 ± 2.35 *μ*m in the control group, mean 10.56 ± 1.01 *μ*m in the statin group, and mean 12.80 ± 0.79 *μ*m in the tetracycline group.

**Figure 15:**
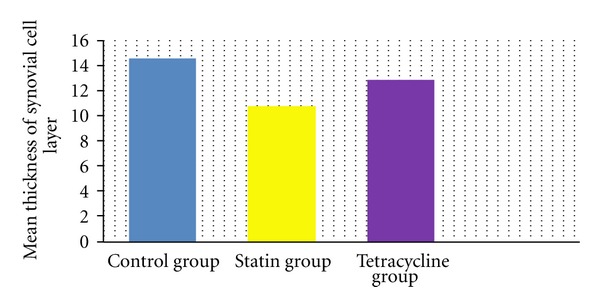
Comparison of the thickness of the cell layer covering the synovia. A statistically significant reduction was determined in the statin group, compared to the control group and the tetracycline group (*P* < 0.05). No significant difference was determined between the tetracycline group and the control group (*P* > 0.05). The results are given as mean points with standard deviation.

Statistical comparison of the results determined a statistically significant difference between the control group and the statin group (*P* < 0.05) and no difference was observed between the control group and the tetracycline group (*P* > 0.05). A comparison between the statin group and the tetracycline determined a statistically significant difference in favour of the statin group (*P* < 0.05) (Figure 15).

## 4. Discussion

 Several different factors have been identified in the pathogenesis of OA [3, 25]. The viscoelastic and compressive properties of cartilage tissue are associated with the ability to protect the structure of the extra-cellular matrix (ECM). In normal cartilage tissue the functions of structuring and destroying the ECM metabolism are a balanced dynamic process. In OA, this balance is disrupted weighted towards destruction, and the destruction itself is caused by increased synthesis and release of catabolic enzymes. Trauma, mechanical stress, or inflammation on the cartilage cells and synovial cells stimulates the expression of cytokines responsible for catabolic activation such as IL-1, TNF-*α*, IL-6, and NO, and the catabolic signal created by these mediators has been shown to activate the matrix metalloproteinases (MMP), collagenase, gelatinase, aggrecanase, elastase, and fibronectin destructive stromelysin-1 which cause cartilage matrix degeneration [3–5]. It has been reported that cartilage degeneration developed by MMP results in intra-articular expression of glycosaminoglycans (GAGs) which then leads to increased cytokines in the synovial fluid and the resultant inflammation which develops in the synovial cells causes reactional synovitis [15, 26, 27].

In recent years, it has been shown that statins as lipid-lowering medication and tetracycline as an antimicrobial agent may have anti-inflammatory and immunomodulator pleiotropic effects on various patient groups and more particularly on different connective tissues [9, 14, 28–30]. Statins have been reported to block MMP expression from vascular smooth muscles by Luan et al. [7], from carotid plaques by Molloy et al. [31], and from gastric epithelial cells by Pillinger et al. [30]. It was determined by Takemoto and Liao [32] that statins were effective in coronary artery disease by blocking the expression of NO in vascular endothelial cells and by suppressing MMP release from macrophages thus preventing the degeneration of artherosclerotic lesions. In a study by Henrich et al. [33] endothelial progenitor cells (EPCs) were isolated from peripheral blood and 5 days of TNF-*α* ve 25 *μ*M simvastatin was administered and apoptosis was determined by TACS-Annexin-V-FITC analysis. It was concluded that the apoptosis of EPC stimulating cells had been blocked by simvastatin and TNF-*α*.

 In studies researching the effects of tetracycline on different connective tissue, intraperitoneal and oral doses varying between 10 and 100 mg have been reported [11, 12, 34]. Studies by Kucuk et al. [11] creating renal reperfusion in mice determined that in the group treated with intraperitoneal 10 mg/kg doxycycline the effect of MMP-2, IL-1, IL-6, and TNF-*α* was significantly reduced and a significant reduction in apoptotic cells was also observed. Iovieno et al. [34] administered 100 mg doxycycline to chronic blepharitis patients for 4 weeks and reported a significant reduction in MMP-9 activity. In an experimental study by Huang et al. [12] where inflammatory colitis was developed in mice, it was shown that the administration of 30 mg/kg intraperitoneal tetracycline blocked iNOS expression in the intestinal tissues, reduced the formation of proinflammatory cytokines and blocked MMP-2, 3, 9, and 13 mRNA transcription.

In the light of these studies, it has been thought that statins and tetracycline may have an effect on connective tissue and cartilage tissue in particular and that they may slow down or prevent the development of OA and there are a limited number of studies related to this [10, 15–20]. The studies which have been carried out have been directed towards the role played by cytokines in the pathogenesis of OA in an in vitro environment and the determination of the MMPs activated by these cytokines. However most of these studies did not examine the macromorphological and histomorphological changes in cartilage tissue wrought by statins and tetracyclines. Therefore, in the current study of an experimental rabbit model, where early-stage osteoarthritis was created by cutting the anterior cruciate ligament, the effects of intra-articular statins and tetracycline on cartilage tissue in vivo were examined macroscopically and microscopically.

 The important role played by matrix metalloproteinases in cartilage degeneration has been shown in several studies [15, 16, 18]. These destructive proteolytic enzymes are produced as the result of stimulated cytokines activating chondrocytes and have been determined to cause the destruction of all the components by attacking the cartilage matrix [16]. Researchers have investigated the role played in OA mechanism by cytokines such as IL-1, IL-6, and NO and the effects of the MMPs activated by them on the molecular level and most studies have been made on a cartilage culture [15, 16, 18]. In a study by Simopoulu et al. [18] of cartilage culture samples obtained from patients with primary osteoarthritis, reverse transcription polymerase chain reaction (RT-PCR) and Western blot analysis were used to evaluate the effects of 2-day administration of 50 *μ*M statin on the production of inflammatory cytokines and proteolytic enzymes and it was reported that this agent had reduced the production of IL-1 and blocked the transcription of MMP-13 mRNA.

In a study by Lazzerini et al. [16] where IL-1 and statin were added to osteoarthritic hip cartilage tissue samples in a culture, incubated for 48 hours, and examined by the ELISA technique, the production of MMP-3 in the chondrocytes was observed to have been inhibited. Leung et al. [35] administered bovine type II collagen intradermally on days 0 and 21 to mice to create arthritis, then from the 21st day, 14 days of intraperitoneal 40 mg/kg simvastatin was given and this agent was seen to have suppressed the collagen-specific Th-1 humoral and cellular immune response, reduced anti-CD3 and anti-CD28 proliferation and inhibited IFN-*γ* expression from mononuclear cells. To show that these effects determined at the molecular level inhibited cartilage tissue degeneration, it was determined in the examinations of cartilage tissue that simvastatin had significantly reduced cartilage surface erosion and GAG loss.

In a study by Akasaki et al. [15] osteoarthritis of the knee was created by cutting the anterior cruciate ligament in a rabbit model and 0.01 mg/mL, 0.1 mg/mL, and 0.5 mg/mL doses of mevastatin were administered intra-articularly as 0.2 mL/kg. Macroscopic and histological examinations of the cartilage tissue layers showed a reduction in full-thickness erosion and subchondral bone erosion and total disorganisation of the cartilage tissue structure and hypocellularity in the layers had been inhibited. Moreover, the results of ELISA and RT-PCR examinations of the synovial tissue showed that by inhibiting monocyte chemotactic protein-1 (MCP-1), IL-1, MMP-3, and MMP-13 gene expression, production had decreased.

Shlopov et al. [17] incubated cartilage tissue samples obtained from total knee replacement patients in a free culture medium for 24 hours, stimulated with TNF-*α*, and 0.1 *μ*g/mL, 10 *μ*g/mL, and 50 *μ*g/mL doxycycline. The results of evaluation by Northern blot and Western blot analyses determined a decrease of 84% on MMP-13 from the 50 *μ*g/mL dose, 72% from 10 *μ*g/mL dose, and only 40% from 0.1 10 *μ*g/mL dose and decreases of 88%, 72%, and 58%, respectively, on MMP-13, thus the conclusion was reached that the effects of doxycycline are dose dependent.

Bowyer et al. [20] evaluated cartilage tissue from guinea pigs by fluoroquinolone assay after 66 days of subcutaneous tetracycline injections and determined 100% decreased activity of MMP-13 and 8, 65% decreased activity of MMP-9, and 24% decreased activity of MMP-1. It was concluded that tetracycline had selective effects on matrix metalloproteinases. As there are no examples of intra-articular administered tetracycline and no studies using the dosage defined in our study, we used a statin dosage similar to that of tetracycline which has been shown to be effective. In the current study, in a developed experimental OA model, using intra-articular administration of statin and tetracycline, the efficacy of which on the production of cytokines and proteolytic enzymes which play a role in the pathogenesis of OA, has been shown in several studies, protective effects were evaluated at a macroscopic and histological level in cartilage and synovial tissue in comparison with a control group.

In the macroscopic evaluation of the efficacy of statin and tetracycline applied intra-articularly, while advanced-stage osteoarthritic changes with subchondral bone and deep layer cartilage tissue erosion were determined in 77% of the control group rabbits, no advanced-stage osteoarthritic changes were determined in the statin group rabbits. In this group only a minimal level of fibrillation was determined in the cartilage tissue lesions. In the tetracycline group, cartilage tissue and subchondral bone erosion (advanced stage) was observed in 50% of the rabbits. In conclusion, statins were determined to have significantly inhibited deep layer erosion and subchondral bone erosion in the macroscopic cartilage tissue structure (*P* < 0.05). However, tetracycline was not seen to have any significant effect on advanced-stage osteoarthritic changes (*P* > 0.05).

 In the histological and histochemical evaluation of the cartilage tissue in this study, when the structural changes of the femoral medial condyle cartilage layer were examined, cleavage to the transitional layer was determined in 33% and full disorganisation in 33% of the control group, while normal cartilage structure was observed in 44% and mild surface disorganisation was observed in 56% of the statin group. In the tetracycline group, cleavage was determined in the transitional, radial, and calcified layer in 60% of the rabbits. In the cellular examination of the cartilage tissue of the control group, 44% cell loss was determined in the tangential layer and 33% cell loss in the transitional and radial layer. In the statin group, the tangential layer was determined as normal in all subjects and only mild hypercellularity was observed in the transitional and radial layers. In the tetracycline group, 60% cell loss was determined in the tangential layer. When tidemark structure and pannus formation were examined, in the statin group pannus had not developed in any of the rabbits and intact tidemark was observed, in the tetracycline group 40% multi level tidemark, and in the control group, 33% indefinite tidemark was determined.

The results of the histological examination of the cartilage tissue in this study determined that statin significantly protected the normal cartilage structure and entirety compared to the control group and there was a significant decrease in cartilage cell loss (*P* < 0.05). No difference was observed between the tetracycline and the control group (*P* > 0.05). In a study by Akasaki et al. [15], statin was administered at 0.2 mL/kg of the highest dose of 0.5 mg/mL and a comparison of this group's findings compared with the control group showed inhibition at rates of 33% in macroscopic degenerative changes, 37% in histological changes, and 34% in synovial tissue changes. In our study, statin was administered at a dose of 0.4 mg/mL/kg and when the results of the statin group were compared with the control group, macroscopic lesions were determined to have been inhibited at a rate of 80%, cartilage tissue histological changes at a rate of 85%, and synovial tissue inflammation at a rate of 65%. The reason for the high rates obtained in this study is thought to be related to the statin dosage.

As has been shown in several previous studies, the macroscopic, histological, and histochemical evaluation of cartilage tissue in our study showed that statins are effective in inhibiting the development of osteoarthritic changes, which is thought to be related to the suprression of the release of these stimulating cytokines (IL-1, IL-6, and TNF-*α*) which play a role in the pathophysiology of osteoarthritis and which activate matrix metalloproteinases (MMP) and at the same time reduce the rate of cell death (apoptosis). Although several studies have shown the protective effects on the development of cartilage tissue lesions, no difference was determined in our study between the tetracycline group and the control group. The reason for the ineffectiveness of this agent may be that the intra-articular application may not be suitable. Also, as has been shown in several previous studies, with the use of different doses of tetracycline in our study, it may be that the effect is dose dependent and the doxycycline used not being a chemically modified agent could have led to these results.

 One of the important molecules providing resistance to compressive loads in the cartilage tissue is glycosaminoglycan [36–38]. Osteoarthritis which develops in the joints is the destruction of aggrecanase with proteoglycan in the cartilage tissue, and an increase in GAG expression has been reported to have been observed. In our study proteoglycan destruction and expression was evaluated with indirect bile-0 involvement. According to this, a mid-severe level of proteoglycan loss of 55% was observed in the control group, while in the statin group the proteoglycan content of the cartilage tissue was determined to have been protected at a rate of 85%. In the tetracycline group a serious level of GAG loss of 40% was determined.

While De Bri and Lei [37] reported from an experimental animal model study that the use of doxycycline was not effective as there was no difference in the GAG content of cartilage tissue between the control group and the subjects that had been treated with doxycycline, Blumberg et al. [36] administered doxycycline for 2 weeks to cartilage tissue obtained from high- and low-energy traumas in a culture medium in an in vitro study and reported from the results that doxycycline was effective in protecting the GAG content of cartilage tissue in the low trauma group but was not effective in the high-energy trauma group.

In our study when the cartilage tissue of the control group and the tetracycline group was compared in respect of GAG content loss, no statistically significant difference was observed (*P* > 0.05). In the statin group, protection of the proteoglycan content of the cartilage tissue was determined as statistically significant compared to the control group and the tetracycline group (*P* < 0.05). It is thought that by inhibiting the production and expression of catabolic matrix metalloproteinases in a certain way, GAG destruction is reduced and thus statins provide protection of cartilage proteoglycan content.

While Akasi et al. [15] showed that statins had not inhibited the production and expression of IL-1, MMP-3, and MMP-13 in cartilage tissue, it was reported that expression of stimulating cytokines and catabolic matrix metalloproteinases (MMP) in synovial tissue had decreased. Borderie et al. [39] applied tetracycline to synovial tissue samples taken from patients undergoing joint surgery and stimulated with IL-1 ve TNF-*α* in a culture environment. The synovial tissues were then examined by fluorometric and immunoblot analysis and the results of the study reported that tetracycline reduced iNOS synthesis and that this effect was dose dependent.

In our study when the synovial tissue histopathological changes and synovial tissue thickness were evaluated, the results determined in the control group moderate-to-severe cell hyperplasia of 77%, moderate-to-severe cell hypertrophy of 77%, and moderate-to-severe level inflammatory cell infiltration of 66%, while in the statin group normal cell structure was determined in 33%, mild hyperplasia in 44%, and inflammatory cell infiltration at a mild level in 33% and at a severe level in 11%.

Evaluation of the tetracycline group determined moderate to severe level cell hyperplasia in 40%, moderate-to-severe level cell hypertrophy in 40% and moderate level of inflammatory cell infiltration in 80%. When subsynovial tissue was evaluated in respect of granulation tissue proliferation, vascularisation, and inflammatory cell infiltration, changes in the statin group were observed to be low compared to the control and tetracycline groups and the changes were determined to be statistically significant and besides the effectiveness of statins on cartilage tissue, the synovial inflammation was observed to have decreased by a significant level (*P* < 0.05). In our study, the inhibition of synovial inflammation by statins, which has been shown in several studies, is thought to have been effective by decreasing the production of catabolic enzymes and MCP-1.

It can be concluded that there is an evident chondroprotective effect of statin. It is thought that the therapeutic effect of this can be increased with some chemical modification for intra-articular application and thus may be a new approach in OA treatment. These effects will be able to be confirmed by in vivo studies together with research at a clinical, macromorphological, histopathological, histomorphometric, and molecular level.

##  Conflict of Interests

It must be stated that the authors do not have any conflicts of interest in this study.
